# An interaction network in the polymerase active site is a prerequisite for Watson-Crick base pairing in Pol γ

**DOI:** 10.1126/sciadv.adl3214

**Published:** 2024-05-24

**Authors:** Joon Park, Geoffrey K. Herrmann, Arkanil Roy, Christie K. Shumate, G. Andrés Cisneros, Y. Whitney Yin

**Affiliations:** ^1^ Department of Biochemistry and Molecular Biology, University of Texas Medical Branch, Galveston, TX 77555, USA.; ^2^ Sealy Center for Structural Biology and Molecular Biophysics, University of Texas Medical Branch, Galveston, TX 77555, USA.; ^3^ Department of Chemistry and Biochemistry, University of Texas at Dallas, Richardson, TX 75080, USA.; ^4^ Department of Pharmacology and Toxicology, University of Texas Medical Branch, Galveston, TX 77555, USA.; ^5^ Department of Physics, University of Texas at Dallas, Richardson, TX 75080, USA.

## Abstract

The replication accuracy of DNA polymerase gamma (Pol γ) is essential for mitochondrial genome integrity. Mutation of human Pol γ arginine-853 has been linked to neurological diseases. Although not a catalytic residue, Pol γ arginine-853 mutants are void of polymerase activity. To identify the structural basis for the disease, we determined a crystal structure of the Pol γ mutant ternary complex with correct incoming nucleotide 2′-deoxycytidine 5′-triphosphate (dCTP). Opposite to the wild type that undergoes open-to-closed conformational changes when bound to a correct nucleotide that is essential for forming a catalytically competent active site, the mutant complex failed to undergo the conformational change, and the dCTP did not base pair with its Watson-Crick complementary templating residue. Our studies revealed that arginine-853 coordinates an interaction network that aligns the 3′-end of primer and dCTP with the catalytic residues. Disruption of the network precludes the formation of Watson-Crick base pairing and closing of the active site, resulting in an inactive polymerase.

## Teaser

Watson-Crick complementary is essential but not sufficient for Pol gamma replication fidelity.

## INTRODUCTION

Mitochondrial genome integrity is essential for cellular energy supplies and metabolism. Accurate replication of mitochondrial DNA (mtDNA) relies on the high-fidelity DNA polymerase gamma (Pol γ) to faithfully copy the parental DNA to the daughter strands. Human Pol γ coordinates the activity of DNA synthesis in its polymerase (Pol) site with self-error correction in the exonuclease (Exo) site to maintain high replication precision (*1*–*3*). Mutations in Pol γ have been implicated in neurological and muscular disorders, and exo-deficient variants lead to premature aging [see review (*4*)].

The accuracy of high-fidelity DNA polymerases is attributed to their ability to select a correct nucleotide from a mixed pool of nucleotides and to proofread any misincorporation. As the correct nucleotide only differs from incorrect ones at the nucleobase, the current view of fidelity is centered on the base selection via Watson-Crick base pairing, and binding to the correct nucleotide induces the obligatory open-to-closed conformational changes to form a catalytic competent *pol* active site. The base complementarity in fidelity is necessary, but is it sufficient in inducing conformational change and reconfiguring the active site?

Pol γ mutations, R853Q and R853W, result in devastating neurological disorders (*5*–*9*). Arg^853^ is not a catalytic residue, yet its substitution for Gln is completely void of polymerase activity (*10*). To investigate the structural basis of the catalytically deficient mutants, we determined a crystal structure of the Pol γ R853A mutant ternary complex with a primer/template DNA and a correct incoming nucleotide, as well as a cryo–electron microscopy (cryo-EM) structure of the wild-type (WT) Pol γ binary complex without bound deoxynucleotide triphosphate (dNTP). The structures revealed an interaction network around Arg^853^ that aligns the catalytic residues with reactants—the 3′-end of the primer and dNTP. The Arg^853^ forms a hydrogen bond with the ribose moiety of dNTP. The mutant Pol γ ternary complex, although bound to the correct dNTP, adopts an open conformation. Our molecular dynamics (MD) analysis showed that Arg^853^ substitution destabilizes the Pol site and alters the energetic landscape of the polymerase. Our results showed that an Arg^853^-coordinated interaction network in the Pol site and its interaction with the ribose moiety are prerequisites for Watson-Crick base pair formation and closing of the active site.

## RESULTS

### Void of polymerase activity in Pol γ R853X mutants

**Fig. 1.**
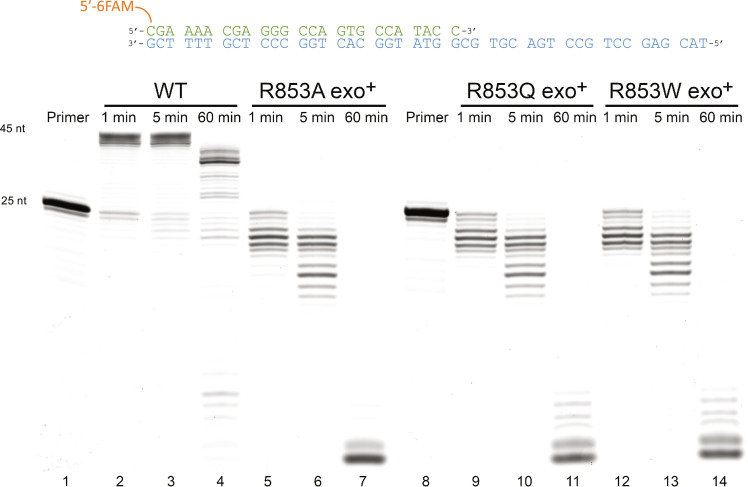
Comparison of polymerase activity between wild-type Pol γ and R853 mutants. Polymerase activity of WT Pol γ (lanes 2 to 4), Pol γ R853A (lanes 5 to 7), Pol γ R853Q (lanes 9 to 11), and Pol γ R853W (lanes 12 to 14) were assayed on 5′-6FAM–25-nt primer (lanes 1 and 8) annealed to 45-nt template at 5, 10, and 60 min.

Pol γ’s catalytic subunit, Pol γA, WT or its variants (R853A, R853Q, or R853W), and WT Pol γB were purified separately to greater than 95% homogeneity and mixed to form a holoenzyme for DNA synthesis assays (Fig. 1). The Pol γ holoenzyme was incubated with equimolar amount of a 5′-6FAM–25-nucleotide (nt) primer annealed to 45-nt template (24 nt/45 nt) duplex (Fig. 1). The reactions were initiated with addition of dNTPs and Mg^2+^. Within 1 min, WT Pol γ extended nearly all primer to the full-length 45-nt product and produced only a trace amount of the shortened primer (Fig. 1, lane 2), whereas R853A, R853Q, and R853W variants failed to produce any synthesis products (Fig. 1, lanes 5, 9, and 12). At 60 min, the full-length 45-nt product was shortened by a few nucleotides in the presence of the WT enzyme, likely due to the slow Exo hydrolysis of the correctly matched 45–base pair double-stranded DNA. In contrast, regardless of the substitution, all Pol γ mutants excised the primer to mostly single and dinucleotides, indicating the lack of Arg^853^ causes a severe imbalance in Pol and Exo activities. The lack of Pol activity is not caused by elevated Exo activity as Pol γ carrying R853A/Q mutants did not exhibit DNA synthesis activity (*10*, *11*).

### Pol γ binary complex structure to complete in a single-nucleotide incorporation cycle

**Fig. 2.**
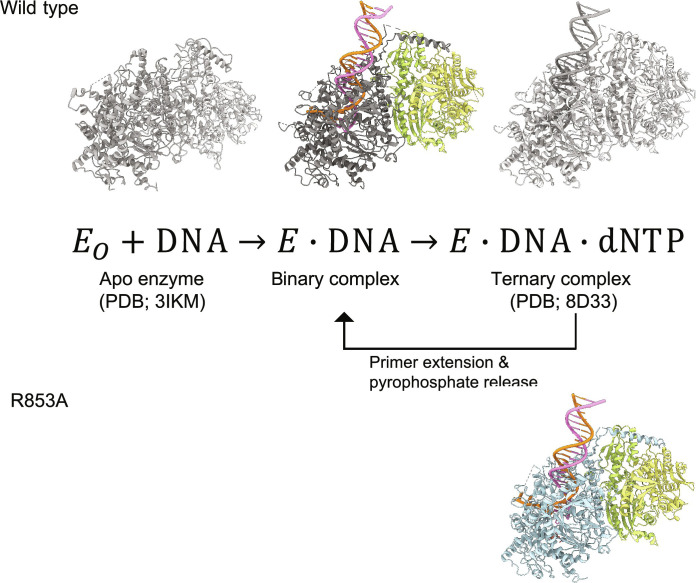
Schematics of nucleotide incorporation cycle with representative Pol γ structures.

To pinpoint the structural defect for Pol γ Arg^853^ substitution reducing the polymerization function, we first completed the structural characterization of a nucleotide incorporation reaction catalyzed by WT Pol γ, which begins with the apo enzyme binding to a primer/template DNA (DNA_n_) to form a binary complex, and then iterates through the three-step cycle: binding of incoming nucleotide in the N-site to form a ternary complex, extending the DNA_n_ primer in the P-site to DNA_n+1_ in the N-site by nucleotidyl transfer reaction, and translocating the newly synthesized primer back to the P-site with subsequent release of pyrophosphate (Fig. 2).

As structures of Pol γ apo enzyme and ternary complex were previously determined (*11*–*13*), we here determined the structure of Pol γ–DNA binary complex using cryo-EM. A homogeneous, a single population was resolved, and the structure was refined to 2.37-Å resolution per estimation by gold-standard Fourier shell correlation (GSFSC) threshold at 0.143 between two independent half-maps (Fig. 2, middle; Table 1; and fig. S1) (*14*). Cryo-EM and crystal structures of Pol γ ternary complexes were previously shown to be identical (*11*), thus comparison can be reliably carried out on structures determined using different techniques.

**Table 1.** Cryo-EM data collection, refinement, and validation statistics of Pol γ binary complex. RMSD, root mean square deviation.

	Pol γ binary complex
**Data collection and processing**	
Magnification	105,000
Voltage (kV)	300
Electron exposure (e^−^/Å^2^)	50
Defocus range (μm)	−1.5–−2.5
Pixel size (Å)	0.425
Symmetry imposed	C1
Number of micrographs	
Collected	8,012
Used	7,137
Initial particle images (no.)	10,947,987
Final particle images (no.)	6,773,711
Map resolution (Å)	
Corrected, FSC = 0.143	2.37
**Refinement**	
Initial model used (PDB code)	8D33
Model resolution (Å)	2.6
FSC threshold	0.5
Map sharpening *B* factor (Å^2^)	
Model composition	
Non-hydrogen atoms	14,676
Protein/DNA residues	1,785/46
*B* factors (Å^2^)	
Protein	36.34
DNA	41.18
RMSD	
Bond lengths (Å)	0.003
Bond angles (°)	0.738
Validation	
MolProbity score	1.12
Clash score	1.27
Poor rotamers (%)	0.19
Cβ outliers (%)	0.00
Ramachandran plot	
Favored (%)	96.02
Allowed (%)	3.92
Disallowed (%)	0.06
*Q*-score	
Protein	0.668
DNA	0.646

The catalytic subunit Pol γA consists of Exo, spacer, and Pol domains. The Pol domain adopts a canonical right-hand configuration, with subdomains termed Thumb that binds to the upstream (−10 to −5 region) of primer/template DNA, Palm that houses the catalytic site, and Fingers that constitutes the incoming nucleotide binding site. The overall structure of the binary complex is similar to the ternary complex [root mean square deviation (RMSD) of 0.713 Å], except the following regions.

#### Opened Fingers

**Fig. 3.**
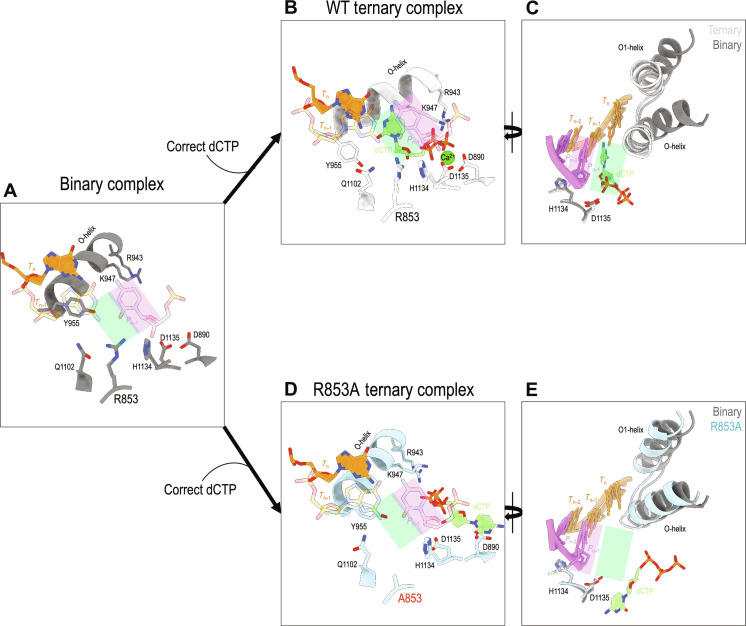
Structural comparison of wild-type Pol γ ternary and binary complexes with Pol γ R853A ternary complex. (**A**) Pol site of WT Pol γ binary complex. (**B**) Pol site of WT ternary complex. (**C**) Superposition of WT Pol γ binary and ternary complexes. (**D**) Pol site of Pol γ R853A ternary complex. (**E**) Superposition of WT binary and Pol γ R853A ternary complexes.

Relative to the dNTP-bound ternary complex, the Fingers subdomain of the binary complex rotates ~30° away from the catalytic center, adopting an “open” conformation (Fig. 3, A and C). If a dNTP was to bind to an open Fingers subdomain in the binary complex, then its α-phosphate (Pα) would be 13.1 Å from the 3′-OH of the primer, a distance too long for phosphodiester bond formation. That distance in the ternary complex is shortened to 4.0 Å. The open-closed Fingers conformational change is critical for the formation of a catalytically competent configuration, which has been observed in other high-fidelity DNA polymerases [see review (*15*)].

#### Pre-catalytic Pol active site

In the binary complex Pol site, Tyr^955^ protrudes into the N-site, and the templating nucleotide *T*_n_ is lifted from the coding-competent position, thus it is unable to form a base pair with the incoming nucleotide. The 3′-end of the primer in the binary complex is in the posttranslocated state in the P-site (Fig. 3A).

In the ternary complex, Arg^853^ and Gln^1102^ are termed the “fidelity switch” (*11*) owing to H-bonding interactions with Watson-Crick paired *T*_n_ and incoming nucleotide as well as the nascent base pair. Arg^853^ also interacts with His^1134^ on the catalytic loop adjacent to the catalytic residue Asp^1135^ (Fig. 3B). All interactions are conserved in the binary complex (Fig. 3A), which holds Asp^1135^ in an identical position in the binary and ternary complexes (fig. S2A).

### Crystal structure of Pol γ R853A ternary complex

To analyze the structural basis of reduced Pol activity and nucleotide binding stemming from Arg^853^ substitution, the ternary complex of the Pol γ mutant holoenzyme was formed with PolγA R853A *exo^−^*; Pol γB ΔI4, a 24-nt/28-nt DNA; and a correct incoming nucleotide, dCTP (2′-deoxycytidine 5′-triphosphate). The complex was crystallized, and diffraction data were collected to 3.4-Å resolution. The structure was determined using molecular replacement using the WT ternary structure as a search model [Protein Data Bank (PDB): 4ZTZ]. The structure was refined to *R*_work_/*R*_free_ = 0.27/0.28 (Table 2).

**Table 2.** Crystal structure refinement statistics of Pol Γ R853a ternary complex.

	Pol γ R853A ternary complex
**Data collection**	
Wavelength (Å)	0.979
Space group	P4_1_2_1_2
Cell dimensions	
*a*, *b*, *c* (Å)	215.41, 215.41, 169.97
α, β, γ (°)	90, 90, 90
Resolution (Å)	48.84–3.435 (3.557–3.435)*
*I*/σ*I*	
Completeness (%)	98.17 (93.98)
**Refinement**	
Resolution (Å)	
Unique reflections	52,823 (4,968)
*R*_work_/*R*_free_	0.196/0.234
No. of atoms	14,330
Macromolecules	14,302
Ligand	28
RMSD	
Bond lengths (Å)	0.004
Bond angles (°)	0.61
Ramachandran	
Favored (%)	96.56
Allowed (%)	3.38
Outliers (%)	0.06
Rotamer outliers (%)	0.00
Clash score	5.03
Average *B* factors	129.73
Macromolecules	126.59
Ligands	219.06

*Statistics for the highest resolution shell are shown in parentheses.

#### Unusual correct nucleotide binding

**Fig. 4.**
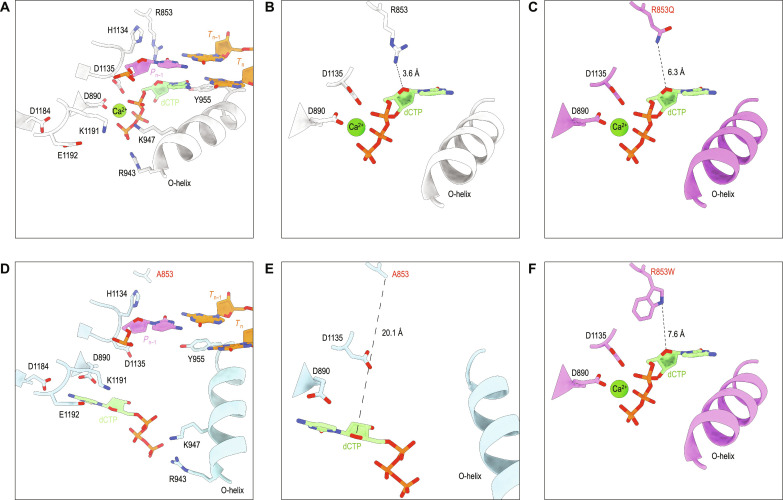
Comparison of dCTP binding between Pol γ wild type and R853A ternary complexes. Polymerase active site of WT Pol γ ternary complex (**A**) and of Pol γ R853A (**D**) ternary complexes. Distance between Arg^853^/Ala^853^ to O4′ of dCTP is shown for WT Pol γ ternary complex (**B**) and of Pol γ R853A (**E**) ternary complexes. Substitutions R853Q (**C**) and R853W (**F**) were made in the Pol γA R853A ternary structure in silico with a minimal clash with surrounding residues in UCSF ChimeraX. The Gln^853^ δNH is 6.4 Å from the O4′ of the dCTP (C). The indole ring of Trp^853^ is unable to form an H bond with the O4′ of the dCTP and its bulky hydrophobic side chain could independently prevent the closing of the Fingers’ domain (F).

Although crystals of the Pol γ R853A ternary complex were formed in the presence of the correct incoming nucleotide, dCTP, the binding mode of dCTP differs from that of the WT ternary complex. In the WT Pol γ ternary complex, dCTP is bound in the N-site by displacing Tyr^955^ and forming base-pairing interaction with the templating base dG_n_; Arg^853^ forms an H bond between its N_η_^+^ and the ribose O4′ of the dCTP (Fig. 3B). In the mutant Pol γ ternary complex, the nucleobase does not form Watson-Crick base pair with the templating dG_n_, instead is rotated ~90°, translated 13.2 Å, and bound in a pocket enclosed by Asp^1192^, Asp^1184^, and Asp^890^, while the triphosphate moiety remains bound to the Fingers domain through charge interactions with Arg^943^ and Lys^947^ (Figs. 3D and 4D and fig. S3). The N-site is still occupied by Tyr^955^, and the templating residue *T*_n_ is precluded from the N-site (Fig. 3D), adopting a configuration nearly identical to the binary complex in the absence of an incoming nucleotide (Fig. 3E).

#### Opened Fingers

Different from the WT ternary complex, the correct dCTP binding did not induce the closing of the Fingers; instead, the Fingers subdomain remains in an open conformation that closely resembles the WT binary complex (Figs. 3, D and E, and 4, A and D); the RMSD of the mutant to the binary complex is 1.435 Å and the ternary complex is 2.248 Å. However, the Fingers subdomain of R853A is slightly less open than the binary complex, with the Fingers subdomain rotated 1.56° toward the active site.

#### Catalytically incompetent Pol site

The loop harboring the catalytic residue Asp^1135^ in the mutant Pol site undergoes a 1.5-Å displacement away from its catalytic competent position (fig. S2B). This misaligned catalytic residue resembles that of the apo enzyme and could, in part, account for the reduced Pol activity (fig. S2C).

Arg^853^ forms a multivalent interaction network with the incoming nucleotide dNTP and nascent base pair. R853A mutation could reduce the affinity of the enzyme to dNTP and subsequently fail to induce Fingers’ conformational change necessary for catalysis, both contributing to the lack of DNA synthesis activity in Pol γ R853X mutants.

### Structural basis for the diseases mutant R853Q and R853W

To understand the disease phenotype of R853Q and R853W, we substituted Ala^853^ in the ternary complex with Gln and Trp, respectively, in silico, with minimal clashes with surrounding residues. The operation shows that R853Q substitution extends the distance between its ε-NH_2_ to ribose’s O4′ of the dCTP to 6.4 Å, more than double the distance of an H bond (Fig. 4C); R853W substitution resulted in a hydrophobic side chain, whose bulky indole could prevent closing of the Fingers domain independently and the N7 of the indole is 7.6 Å from the O4′ of the dCTP (Fig. 4F). Thus, R853Q and R853W substitutions share the same defects as the R853A, where the H-bonding network coordinated by R853 with the dNTP and with the catalytic triad are abolished.

### Incoming nucleotide binding affinity

**Fig. 5.**
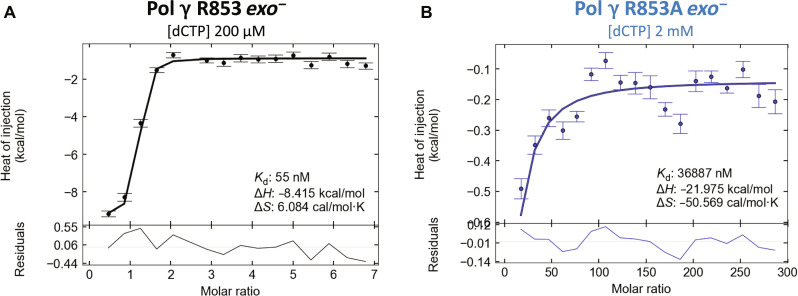
Isothermal calorimetry data of correct incoming nucleotide binding in Pol γ. ITC graphs of WT Pol γ (**A**) and Pol γ R853A mutant (**B**) binary complexes binding to dCTP.

To independently verify the structural conclusion that Arg^853^ substitution reduced dNTP binding affinity, we used isothermal titration calorimetry (ITC) to measure a correct incoming nucleotide (dCTP) binding to Pol γ WT *exo^−^* and R853A *exo^−^* (Fig. 5). The dCTP was titrated into a preformed binary complex of Pol γ and 25-nt/45-nt DNA up to 200 μM and 2 mM for WT (Fig. 5A) and R853A (Fig. 5B), respectively. The measured dissociation constants (*K*_d_) were 55 nM for WT and 37 μM for R853A, which correspond to ΔG = −10.329 for WT and −6.298 kcal/mol for R853A, indicating that the lack of Arg^853^ resulted in ~4 kcal/mol binding energy reduction and a 670-fold lower binding affinity.

### Global and domain motion changes revealed by molecular simulations

As the biochemical and structural analyses revealed an imbalance in Pol and Exo activities and the lack of necessary conformational changes in the R853A mutant, we evaluated the difference between the mutant and WT Pol γ using MD simulations. Note that the initial point of the simulation of the two structures is the same, namely, the WT Pol γ closed, ternary complex (PDB: 4ZTZ) except for the substitution at residue 853 for the mutant system. During the visualization of the simulation, we noticed that the regions modeled by Rosetta exhibited high flexibility, thus increasing the overall average RMSD of the system, but the domains present in the crystal structures were unaffected. Therefore, the analyses only included specific subdomains on Pol γA (Palm, Fingers, and Thumb) present in the crystal structure and excluded the homology-modeled region. The calculations have been carried out in triplicate, the results for all three replicates for each system are similar, and thus, the first replicate has been chosen as representative for the discussion below.

**Fig. 6.**
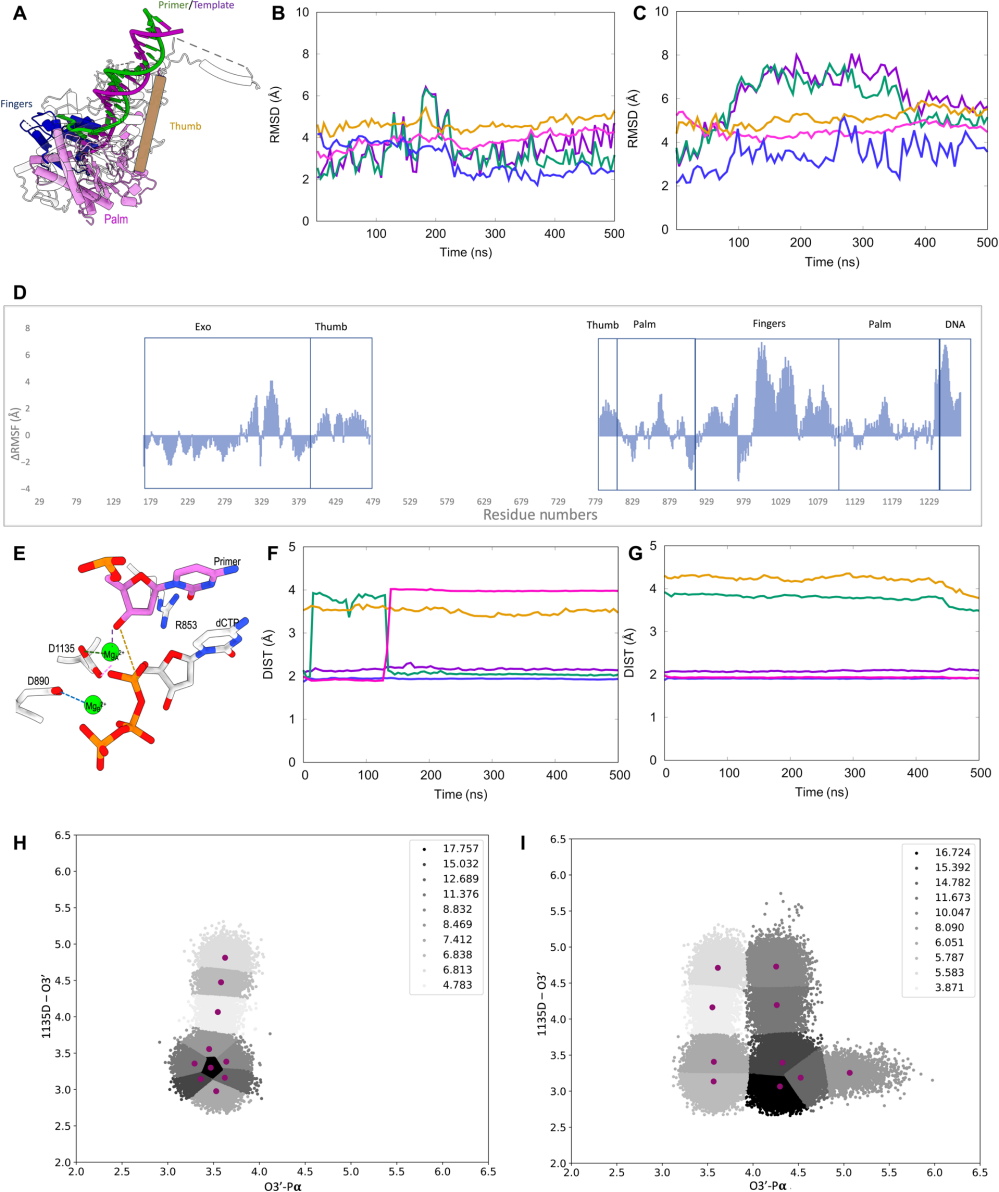
Molecular dynamics simulation of wild-type Pol γ and Pol γ R853A ternary complexes. (**A**) Structural subdomains of Pol γA: pink, Palm; tan, Thumb; blue, Fingers; green/purple, DNA primer/template. RMSD per subdomain for WT Pol γ (**B**) and Pol γ R853A mutant (**C**) following the color scheme in (A). (**D**) Difference between the average values of RMSF between Pol γ R853A mutant and WT pol γ (Mut-WT). (**E**) Polymerase active site with representative catalytic distances denoted by dashed lines. Color key: purple, Mg_A_^+2^ and O3′ of DNA primer; tan, O3′ and α-phosphate of dCTP; green, OD2 of Asp^1135^ and Mg_A_^+2^; pink, OD1 of Asp^1135^ and Mg_A_^+2^; blue, Asp^890^ and Mg_A_^+2^. Distance analysis of the WT Pol γ (**F**) and the Pol γ R853A mutant (**G**) for the catalytic residues in the polymerase active site following the color scheme in (E). *k*-means clustering analysis for the WT Pol γ (**H**) and Pol γ R853A (**I**) based on the O3′–α-phosphate (*x* axis) and the Asp^1135^–O3′ (*y* axis) distances. Replicas of these calculations are presented in fig. S9 (A to F).

The average RMSD for the DNA bound to the mutant is notably higher than that to WT, and the mutant’s Thumb, Fingers, and Palm subdomains also exhibit increased RMSD in the mutant compared with the WT, albeit at lower values (Fig. 6, B and C; tan, blue, and pink lines, respectively), indicating a deviation from the WT structure. The primer/template in the mutant exhibited the largest deviation from the WT (Fig. 6, B and C; green and purple lines), suggesting that the mutation affects the configuration of the bound DNA. Similarly, the average root mean squared fluctuation (RMSF) shows that the above subdomains exhibit larger fluctuations in the mutant than in the WT (Fig. 6D). The average RMSF of the Exo domain was observed to be higher in the WT than in the mutant.

A dynamic cross-correlation analysis was done to investigate the effect of the mutation on the correlated motion of the systems (fig. S4). Difference plots between the mutant and the WT, with the WT as a reference, show several changes in correlated movement between the domains of the polymerase for the two systems (fig. S4A). Most notably, the Fingers subdomain of the polymerase shows an increased anticorrelated movement with the Thumb and Palm subdomains in the WT system as compared to the mutant (fig. S4B). This indicates that the residues in the Fingers subdomain move in an opposite direction to that of the Thumb and Palm subdomains in the WT, indicating the possibility of an opening and closing motion of the polymerase in the WT, while this motion is not observed in the mutant system. The Exo domain and the Fingers subdomain show an increased correlation in the mutant as compared to the WT. As the Exo domain remains static during the reactions, the results suggest a reduced likelihood for the Fingers subdomain to undergo the closing conformational change.

The essential dynamics of the domains are better illustrated by the normal mode analysis (NMA) (fig. S5). The top 10 modes among the 100 modes calculated were considered. Two types of motion were seen to be the primary contributors to the dynamics of the systems: breathing and rocking (movie S1). The primary contributor for the WT system, mode 1 (around >95%) corresponds to a breathing-like motion with most changes observed on the Thumb, Fingers, and Palm subdomains, while the second mode (<5% contribution to overall motion) corresponds to a rocking motion (fig. S5, A, C, and E to G; and movie S1). Conversely, for the mutant system, the first mode corresponds to the rocking motion and represents about 60 to 70% of the overall contribution to the motion, and mode 2 has a much higher contribution, around 20 to 40% in the mutant, and corresponds to a breathing motion (fig. S5, B, D, and H to J; and movie S1). It is interesting to note that the contributors to the motion are inverted from the WT to the mutant. The breathing motion of the WT is also consistent with the dynamic cross-correlation difference analysis in the previous section, where it was seen that, in the WT, the Fingers show obvious anticorrelated movement with the Thumb and Palm subdomains, suggesting an opening and closing movement of the system in the WT, similar to a breathing motion, which is not the dominant movement in the mutant. These results suggest that the WT system exhibits dynamic motion that is consistent with Pol activity since the breathing motion corresponds to the natural open-to-close motion observed for the binary to ternary transition. On the other hand, the mutation changes the dynamics of the system to a more rigid rocking motion, markedly reducing the contribution from the breathing motion, which suggests an inhibition of the motion of the polymerase, hence causing a disruption to the catalysis.

### Indication of catalytic incompetent Pol site in R853A by MD simulation

We sought to map the R853A substitution-induced changes in the Pol site using MD simulations (Fig. 6, E to G). We initially focused on four key distances: (i) Primer O3′ to dCTP Pα (tan), (ii) Primer O3′ to Mg_A_^2+^ (purple), (iii) Asp^1135^ OD_δ_ to Mg_A_^2+^ (green and pink), and (iv) Asp^890^ OD_δ_ to Mg_B_^2+^ (blue). The main difference in distance was between the Primer O3′ and the dCTP Pα, which extends from 3.5 Å in the WT to 4.3 Å in the R853A mutant (Fig. 6, F and G; tan line).

A clustering analysis using the *k*-means algorithm was performed, considering all calculated structures (combined trajectories = 150,000 snapshots; see the Supplementary Materials for clustering based on individual replicates). Distances of the Primer O3′ to the dCTP Pα (d1) were plotted against that of the Primer O3′ to Asp^1135^ (d2) for WT and R853A mutant (Fig. 6, H and I). In the WT, 40% of the snapshots corresponding to three clusters are within d1 = {3.0 to 3.8 Å} and d2 = {2.8 to 3.5 Å} (Fig. 6H), whereas less than 15% of the snapshots in the mutant (corresponding to only one cluster) were located in the same area (Fig. 6I). Moreover, most of the snapshots in the R853A mutant structure are clustered at longer d1 and d2 distances, which is consistent with an open Fingers position resulting in an extended distance between the reactants for DNA synthesis.

A superposition of WT crystal structure (PDB: 4ZTZ) (fig. S6A, green) and a representative of the WT simulation (fig. S6A, gold) show high similarity (fig. S6A), whereas R853A simulations (fig. S6B, pink) shows that the substitution resulted in an open Fingers and Thumb configuration that are deviated from the WT crystal structure (fig. S6), with the RMSD values for the Thumb and Fingers between the crystal structure and the mutant MD representative at 7 and 4 Å, respectively. Together, these results show that the MD simulations reproduce the observed experimental structural features and provide further insights into the major impact of the R853A mutation on the structure of Pol γ.

We next analyzed residues involved in stabilizing or destabilizing dCTP (fig. S7A) and the mutation site at Arg^853^ (fig. S7B) using energy decomposition analysis (EDA). Residues with the largest energetic contribution are listed (fig. S7C). Arg^853^ provides the largest, 123.8 kcal/mol, stabilization energy to dCTP in the WT, whereas Lys^1191^ is the largest contributor 125.0 kcal/mol to dCTP in mutant Ala^853^ structure (fig. S7, A and C). Arg^853^ mutually stabilizes Asp^890^, Glu^895^, His^1134^, Asp^1135^, and Glu^1136^, forming an interaction network. Substitution of Arg to Ala loses 123 kcal/mol stabilization energy, which is compensated by energetics from Arg^866^, Asp^890^, Arg^943^, Lys^947^, Lys^1191^, and Glu^1136^. The computational results are in good agreement with the mutant ternary structure where the nucleobase of dCTP binds to a pocket of Asp^890^, Lys^1191^, and Glu^1192^, while the triphosphate is bound to Arg^943^ and Lys^947^. To account for the 50,000 structures used in each replicate, the EDA of representative replicates of both WT and mutant systems have been presented with error bars, as separated Coulomb and van der Waals interactions, with mutation site Arg/Ala^853^ as the reference residue (fig. S8). The small error bars show that the interactions between the residues remain mostly similar throughout the simulation.

## DISCUSSION

Mitochondrial genome integrity is essential for the organelle functions. Mutations on mtDNA are associated with multi-system disorders. We investigated Pol γ Arg^853^ mutations (R853Q and R853W) that are implicated in severe clinical disorders (*8*, *9*). Although not a catalytic residue, substitutions of Pol γ Arg^853^ are completely void of polymerase activity. Our studies revealed that Pol γ achieves high fidelity by a balanced energy partition for the moieties of a correct nucleotide, which could be generalized to other A-family DNA polymerases due to their shared structural features.

### Modular energetics of dNTP binding

To maintain high replication fidelity, a DNA polymerase should not only select a correct nucleotide from the pool deoxy- and ribonucleotides but also recognize chemical modifications to its nucleobase, deoxyribose, and triphosphate moieties. A high-fidelity DNA polymerase uses checkpoints for selection of the correct nucleobase via Watson-Crick base pairing, as well as base-shape complementarity because even modified nucleotides without H bonding also can be incorporated (*16*), albeit at much lower efficiency; selection of the deoxyribose moiety against ribose is accomplished by a “steric gate” tyrosine (*17*, *18*), and selection of the triphosphate moiety is likely done by its configuration (*19*). Proofreading of misincorporation in the Exo site further increases the replication fidelity (*16*, *20*, *21*).

In this study, we identified Arg^853^ in Pol γ binds to the deoxyribose of dNTPs. Substitutions of Arg^853^ (R853Q/W/A), conserved among A-family DNA polymerases, eliminate this interaction, markedly reducing dNTP binding affinity. We noticed that the ~10 kcal/mol of free enzyme for Pol γ binding to a correct nucleotide is contributed modularly by the interaction with dNTP moieties: the electrostatic interaction with triphosphate at the initial binding contributes 4 to 5 kcal/mol (*22*), and Watson-Crick base pairing contributes ~0.3 kcal/mol free energy (ΔΔ*G*) between correct and incorrect nucleobases (*23*). The energetic discrepancy suggests additional contributors to dNTP binding energy. In this study, we identified that Pol γ Arg^853^ forms an H bond with the ribose moiety of the incoming nucleotide, the measured ΔG difference between WT and R853A mutant for dCTP binding is 4 kcal/mol, or about 40% of total dNTP binding, which is greater than a single hydrogen bond, showing additional energetic contribution of the Arg^853^ beyond interaction with the ribose. Nevertheless, the ribose interaction enables the polymerase to distinguish deoxyribose from other pentocyclic analogs, such as cyclopentane, thiophene, or selenophene.

Despite the moieties contributing unequally to the free energy to enzyme binding to dNTP, missing any of the components will prevent dNTP incorporation and halt the polymerase DNA synthesis. The formation of Watson-Crick base complementarity is essential but additional interaction with the ribose and phosphate moieties is sufficient for a correct nucleotide incorporation by Pol γ. Only when all moiety checkpoints are satisfied does the polymerase incorporate the dNTP. The modular dNTP recognition is not only critical to mtDNA replication fidelity but also important for Pol γ exclusion of nucleotide/nucleoside antiviral drugs and reducing drug toxicity.

### The central role of Arg^853^ in the catalytic interaction network in the Pol site

**Fig. 7.**
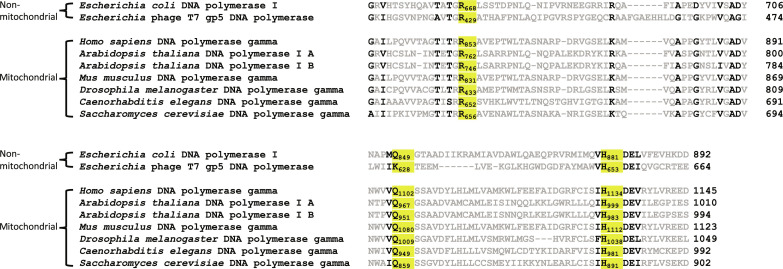
Sequence alignment of replicative A-family DNA polymerases. Sequence alignment of Pol γ with other non-mitochondrial (top) and mitochondrial (bottom) A-family DNA polymerases. Highly conserved amino acids are shown in black, and the “fidelity switch” (Arg^853^, Gln^1102^, and His^1134^ in Pol γ) has been highlighted in yellow.

Our structural studies revealed an interaction network in Pol γ Pol site centered at Arg^853^ that stabilizes the two reactants of phosphoryl transfer reaction, the 3′-end of the primer DNA and dNTP and coordinates the catalytic residue Asp^1135^ via His^1134^. The Arg-His-Asp residues are highly conserved both in sequence and in structure among members of A-family DNA polymerases that include *Escherichia coli* DNA Pol I, T7 DNA Pol, and most mtDNA polymerases (Fig. 7).

Mutation studies of the Pol I equivalent Arg^668^-His^881^-Asp^882^ residues revealed their contribution to the enzyme kinetics: Arg^668^ mutation reduced the catalytic proficiency by 1300-fold with both increased *K*_m_ and reduced *k*_cat_, whereas mutations on Asp^882^ or His^881^ only reduced the *k*_cat_ by ~400- and 10-fold, respectively. Consequently, the Arg^668^ mutant reduced the catalytic proficiency more than the catalytic residue Asp^882^ (*24*–*26*). The kinetic studies are in good agreement with structural and functional studies of Pol γ where Arg^853^ mutations resulted in an open, catalytically incompetent configuration upon binding to a correct dNTP with shifted catalytic loop. The importance of Arg in the coordination of the 3′-end of the primer and dNTP was also tested in DNA Pol I. When substrate dNTP was substituted with dcUTP, where the O4′ of the ribose was replaced with a C4′ and the H bond between Arg^668^ with the ribose would be obliterated, the *k*_pol_ was reduced by 1600-fold, similar to the magnitude of reduction in abolishing the H bond between Arg^668^ and 3′-OH of the primer (*27*).

MD simulations for the Pol γ R853A revealed two major impacts of the in silico mutation: (i) elongated distance between the 3′-OH of primer DNA and Pα of dCTP and (ii) higher flexibility of the Fingers subdomain relative to other subdomains. The changes are consistent with the noncanonical binding of dCTP to open the Fingers subdomain in Pol γ R853A crystal structure. Such abnormal dCTP binding mode loses Watson-Crick base pair interaction and is seen interacting with a protein pocket, which is in excellent agreement with EDA analysis showing that the same residues selectively stabilize R853A substitution. Considering MD simulations for the Pol γ R853A variant started from a catalytically competent, closed WT ternary conformation, the results revealed intrinsic functions of Arg^853^ that deviate from the starting WT structure, providing strong independent support to the experimental R853A ternary complex structure.

### Altered enzyme dynamics by Arg^853^ mutation

MD simulation and NMA indicate that Pol γ R853A displays dynamic changes from the WT. While wild-type motion is predominantly in a “breathing” mode, which likely is a movement necessary for proper catalysis, a smaller population (20 to 40%) of Pol γ R853A adopts such mode, suggesting that catalytically competent movement (closing of the Fingers) is still possible albeit less efficient. The *k*-means clustering analysis supports such an idea, as Pol γ R853A shows a bimodal distribution: while a large population is catalytically incompetent (d1 > 4.0 Å), a small fraction of them maintains the catalytically competent distance (d1 < 3.8 Å), similarly to that of the WT. However, as evident by studies in this work and those in the literature, correct configuration alone is insufficient for catalysis, and the presence of Arg^853^ in Pol γ is an absolute necessity.

We noticed that the distance between the C_β_ of Ala^853^ and O4′ of dCTP in the Pol γ mutant ternary complex crystal structure is longer than that in the MD simulation. The observation, together with increased dynamics induced by the mutation, suggests that the crystal structure likely represents one conformation in a dynamic ensemble of populations. Probing the mutational effect on Pol γ dynamics warrants further investigation.

## MATERIALS AND METHODS

### Protein expression and purification

Proteins were prepared as previously described (*13*). Briefly, Pol γA WT, Exo-deficient [D198A/E200A (*exo^−^*)], and mutants [Pol γA R853A, Pol γA R853Q, Pol γA R853W, and Pol γA R853A (*exo^−^*)] were expressed in insect cells Sf9 and purified using TALON (Clontech) and Superdex 200 (Cytiva) column chromatography. Pol γB WT and a deletion mutant Pol γB-ΔI4 (deletion of amino acids 136 to 182) were expressed in *E. coli* Rosetta BL21 DE3 and purified using Nickel-NTA (Qiagen) and cation-exchange (Mono S) chromatography. For structural studies, purified Pol γA and Pol γB were mixed at a 1:2 molar ratio on ice for 10 min and purified using Superdex 200 (Cytiva) column chromatography. The protein purity was estimated to be >95% per SDS–polyacrylamide gel electrophoresis analysis.

### Oligonucleotide substrates

For activity assays, a 25-nt primer (5′-CGA AAA CGA GGG CCA GTG CCA TAC C-3′) with 5′-6FAM modification and a 45-nt template (5′-TAC GAG CCT GCC TGA CGT GCG GTA TGG CAC TGG CCC TCG TTT TCG-3′) were synthesized by Integrated DNA Technologies. Primer and template were annealed at a 1:1 ratio in buffer [20 mM tris (pH 8.0), 100 mM NaCl, and 1 mM EDTA (pH 8.0)] by heating the mixture to 95°C for 5 min, followed by slow cooling to room temperature overnight.

High-performance liquid chromatography–purified 24-nt primer (5′-CGA AAA CGA CGG CCA GTG CCA TAC-3′) and 28-nt template (5′-CGA GGT ATG GCA CTG GCC GTC GTT TTC G-3′) were synthesized and annealed as described above for crystallographic and cryo-EM studies. For crystallization, dC at the 3′-end of the 24-nt primer was replaced with ddC to prevent any enzymatic reaction.

### Polymerase activity assays

A total of 400 nM Pol γ and 400 nM duplex DNA were mixed on ice in reaction buffer [25 mM Hepes (pH 7.5), 140 mM KCl, 5% glycerol, 1 mM EDTA (pH 8.0), and BSA (100 μg/ml)] and prewarmed at 37°C for 5 min. Reactions were initiated by adding an equal volume of reaction buffer containing 20 mM MgCl_2_ and 100 μM dNTP. Final concentrations were 200 nM for Polγ-DNA complex, 10 mM for MgCl_2_, and 50 μM for dNTP. Reactions were incubated at 37°C for the indicated time and stopped by adding the fourfold excess volume of quench buffer [80% formamide, 50 mM EDTA, 0.1% (w/v) SDS, 5% glycerol, and 0.02% bromophenol blue]. Reaction products were denatured by heating at 95°C for 5 min, resolved on a 20% polyacrylamide denaturing (7 M Urea) gel electrophoresis, and imaged using an Amersham Typhoon Biomolecular Imager (Cytiva).

### Isothermal titration calorimetry

Pol γ WT *exo^−^* and R853A *exo^−^* were dialyzed against Buffer RX [25 mM Hepes (pH 7.5), 140 mM KCl, 10 mM CaCl_2_, 5% glycerol, and 1 mM β-mercaptoethanol (BME)] at 4°C overnight. dCTP was diluted from a 100 mM stock to the indicated concentration. Following dialysis, protein concentration was determined using the extinction coefficient ε_280_ = 3.86 × 10^5^ M^−1^ cm^−1^ for the heterotrimer. Pol γ:DNA complexes were made by incubating protein and DNA at a 1:1 ratio (5 μM:5 μM) at room temperature for 10 min. Titrations were carried out using a Malvern MicroCal PEAQ-ITC at 37°C with 19 injections (0.4 μl for the first injection, followed by 18 injections of 2 μl) of 200 μM or 2 mM dCTP titrated into 5 μM protein:DNA complex. Control titrations were performed by titrating 200 μM or 2 mM dCTP into the buffer. Thermodynamic binding parameters were determined using NITPIC and SEDPHAT as described in (*28*).

### Crystal structure determination of Pol γ R853A ternary complex

#### Crystallization

For crystallization, 63.5 μM complex PolγA R853A (*exo^−^*)/PolγB ΔI4 was mixed with 114.3 μM 24-nt/28-nt DNA in the presence of 10 mM CaCl_2_ and 2 mM dCTP and subjected to crystallization after incubation on ice for 10 min. Crystals were formed using the hanging-drop vapor diffusion method at 20°C against the well solution [100 mM MES (pH 6.0), 150 mM NaCl, 10 mM CaCl_2_, 50 mM BME, 3% PEG-8000 (polyethylene glycol, molecular weight 8000), 1 to 8% sucrose, 2% Jeffamine, and 0.8 M nondetergent sulfobetaine (NDSB)]. Crystals were cryoprotected in solutions containing 30% glycerol, 30% 2-Methyl-2,4-pentanediol (MPD), 25 mM BME, and 1 mM dCTP and flash-frozen.

#### Data collection, processing, and refinement

Diffraction data were collected from a single crystal at beamline 5.3 Advanced Light Source (ALS, Berkeley Laboratory) using wavelength 1.0-Å x-ray, recorded on a charge-coupled device detector. The crystals exhibited a tetragonal space group, P4_1_22, with cell dimensions *a* = 215.41, *b* = 215.41, and *c* = 169.97. Data were processed with HKL3000 (*29*). The structure was determined using molecular replacement using a WT Pol γ ternary complex (*12*) as the search model. The Fingers movement in the mutant structure was clearly valuable in the Fo-Fc map and was manually built into 2Fo-Fc and Fo-Fc maps using Coot (*30*). The structure was refined using PHENIX (*31*).

### Cryo-EM structural determination of wild-type Pol γ binary complex

#### Sample preparation

WT Pol γ (2 μM) was mixed with 2 μM DNA and incubated on ice in a buffer containing 20 mM Hepes (pH 7.5), 140 mM KCl, 1 mM EDTA (pH 8.0), 10 mM CaCl_2_, 10 mM BME, and 0.01% (w/v) octyl-β-glucoside. A frozen grid was prepared by applying 4 μl of the sample on a plasma-cleaned QUANTIFOIL R 2/1 Cu 200 grid (Electron Microscopy Sciences) using Vitrobot Mark IV at 22°C and 100% humidity (Thermo Fisher Scientific).

#### Data collection

Frozen grids of Pol γ binary complex were loaded into a Titan Krios G3i (Thermo Fisher Scientific) equipped with K3 direct electron detector with BioQuantum energy filter (15-eV energy slit) (Gatan) and operating at 300 keV at Stanford SLAC Cryo-EM Center. Cryo-EM data were automatically acquired using EPU software in counted super-resolution mode at a nominal magnification of ×105,000 (corresponds to 0.43 Å/pix) with a nominal defocus range between −1.5 and −2.5 μm. Forty-frame movie stacks were collected over 2-s exposure with a total dose of 49.6 e^−^/Å. A total of 8012 movie data were collected.

#### Structural determination

The movie frames were imported into cryoSPARC (*32*) for image processing (fig. S1A). Movies were motion-corrected and 2× binned using Patch Motion Correction. Contrast transfer function (CTF) of resulting micrographs was determined using patch CTF estimation. After preprocessing and discarding micrographs with CTF fit higher than 4 Å and a total motion less than 50 pixels, 10,947,987 particles were picked from the resulting 7137 micrographs using Blob Picker. Particles were extracted with 4× binning (80 pixels) and subjected to two iterative rounds of 2D classifications. A total of 200,000 particles out of the cleaned 6,773,711 particles were used in the Ab Initio building of three initial volumes. All particles were subjected to heterogeneous refinement using three volumes from Ab Initio (classes 1, 2, and 3). Then, 3,342,218 particles belonging to class 1 were subjected to another round of heterogeneous refinement using identical input volumes. A total of 2,732,032 particles in class 1 from the second heterogeneous refinement were recentered and re-extracted without binning (320 pixels), resulting in 2,504,778 particles. Homogeneous refinement of 2,504,778 particles resulted in 2.47 Å. Particles were further separated on the basis of conformational heterogeneity by running another round of heterogeneous refinement with three repeated input volumes resulting from the homogeneous refinement. Particles belonging to classes 1–1 and 1–3 were combined and further refined by homogeneous refinement followed by cryoSPARC’s implementation of local and global CTF refinements (*33*) and a final round of non-uniform refinement (*34*), yielding 2.37-Å reconstruction according per GSFSC at 0.143 (fig. S1B). Local resolution of final reconstruction was estimated by cryoSPARC’s implementation of blocres (*35*) and colored by resolution range from 2 to 4 Å in UCSF ChimeraX (fig. S1B) (*36*).

The cryo-EM map was postprocessed with LocSprial (*37*). Cryo-EM structure of Pol γ–DNA–dCTP ternary complex (PDB ID: 8D33) (*11*) was rigid body fitting into LocSpiral-processed map using UCSF Chimera (*38*) and the unfitted regions were manually adjusted using Coot. The model was refined using real-space refinement in PHENIX (*31*), Coot (*39*), and ISOLDE (*40*). The refined model was validated by MolProbity (*41*) and *Q*-score analysis (*42*). Figures were prepared using UCSF Chimera (*38*) and UCSF ChimeraX (*36*).

### Molecular dynamics simulations

#### Preparation of structural model for simulation

The crystal structure of mtDNA polymerase γ (4ZTZ) was used as the initial model. The missing regions were completed using homology modeling via RosettaFold (*43*). Twenty-five structures were built using Rosetta, and then they were compared to the crystal structure by overlaying the Rosetta structure with the crystal structure. Ten structures were selected from the best RossettaFold solutions and visually inspected. Each of the 10 structures was loaded onto visual molecular dynamics (*44*), and the RMSD with respect to the regions resolved in the crystal structure was calculated. The structure with the lowest overall RMSD (2.5 Å) was selected as our initial system. The mutated system was created by in silico introducing Arg^853^ to Ala substitution using Chimera UCSF (*38*).

#### MD simulation

The parameters for the incoming nucleotide in the Pol γ WT and R853A ternary complexes were set as previously reported (*45*). The protonation states were determined using ProPKA (*46*) and hydrogens were added to amino acids using MolPobity (*41*). The LeAP (*47*) module of AMBER18 was used to neutralize and solvate the system. The AMBER ff14SB (*48*) and gaff (*49*) forcefields were used for the protein and OL15 (*50*) for the DNA. The system was neutralized using 29 Mg^+2^ ions and the system was solvated using the TIP3P water model in a cubic box with a minimum of 12 Å between the edge of the protein and the box. Then, the volume of the system was noted, and 73 Mg^2+^ and 146 Cl^−^ ions were calculated to make the system into a 20 mM solution of MgCl_2_. Once the number of ions was determined, they were added at random to the system. The final system has 239,364 atoms for the WT and 236,912 atoms for the mutant.

The MD simulations were run using AMBER18 pmemd.cuda (*51*). Minimization was performed for a total of 10,000 cycles, with 5000 cycles using the steepest descent algorithm and the other 5000 cycles using the conjugated gradient algorithm followed by heating to 300 K using Langevin dynamics (*52*) with a collision frequency of 2 ps^−1^. During this process, all heavy atoms were restrained with a force of 100 kcal mol^−1^ Å^−2^. Subsequently, the restraints were reduced gradually (table S1).

After the release of restraints, it was observed that some distortion occurred around the active site; therefore, selected residues in the Pol site, namely, the incoming nucleotide, Asp^890^ and Asp^1135^, Mg_A_^2+^ and Mg_B_^2+^, and the primer 3′-OH terminus were restrained with a 0.5 kcal mol^−1^ Å^−2^ force for an additional 25 ns. The restraint was removed after 25 ns and the remainder of the simulations were run without restraints. Once equilibrated, both systems were subjected to 500-ns NPT MD in triplicate with a time step of 2 fs, resulting in a total of 1.5 μs for each of the systems. All bonds involving hydrogen atoms were treated using SHAKE (*53*) and long-range Coulomb interactions were handled with the smooth particle mesh Ewald method using a 10-Å cutoff for non-bonded interactions.

#### Trajectory analysis

The CPPTRAJ (*54*) module of AMBER18 was used to analyze the production trajectories. These analyses include the RMSD and correlation matrices. NMA was performed using ProDy (*55*). Various Python and R libraries were used to organize and analyze the raw data and Gnuplot (*56*) and Matplotlib (*57*) were used to put the data into a graphical format. EDA can be used to calculate the non-bonded interactions (Coulomb and van der Waals) to qualitatively assess the stabilizing or destabilizing role of different amino acids in a variety of protein systems (*58*–*60*) both for MD and QM/MM calculations. Amber-EDA was used to calculate these interactions with respect to specific residue sites using an in-house Fortran90 code (*61*). For this study, the analyses were run in three replicates of WT and the R853A mutant, and the results were averaged over the three systems. The residues chosen as reference were the incoming nucleotide and the mutation site Arg/Ala^853^. The protein system was separated into Palm, Fingers, and Thumb domains and the structural analyses were only done on the domains present in the crystal structure so that the extra flexibility from the modeled loops do not influence the data.

### Experimental replicas

Activity assay and MD simulations were repeated three times. The results of each replicate of the MD simulations are shown in fig. S11.
