# Supplementary material for: An interaction network in the polymerase active site is a prerequisite for Watson-Crick base pairing in Pol γ
Source: Sci Adv. 2024 May 24;10(21):eadl3214. doi: 10.1126/sciadv.adl3214 (PMC11122685; doi:10.1126/sciadv.adl3214)
Supplement: Supplementary file 1 — Figs. S1 to S9 Table S1 Legend for movie S1 [file sciadv.adl3214_sm.pdf]

Science Advances
AAAS

Supplementary Materials for

An interaction network in the polymerase active site is a prerequisite for Watson-Crick base pairing in Pol γ

Joon Park *et al.*

Corresponding author: G. Andrés Cisneros, andres@utdallas.edu; Y. Whitney Yin, ywyin@utmb.edu

Sci. Adv. **10**, eadl3214 (2024)
DOI: 10.1126/sciadv.adl3214

The PDF file includes:

Figs. S1 to S9
Table S1
Legend for movie S1

Other Supplementary Material for this manuscript includes the following:

Movie S1

Supplementary Figures

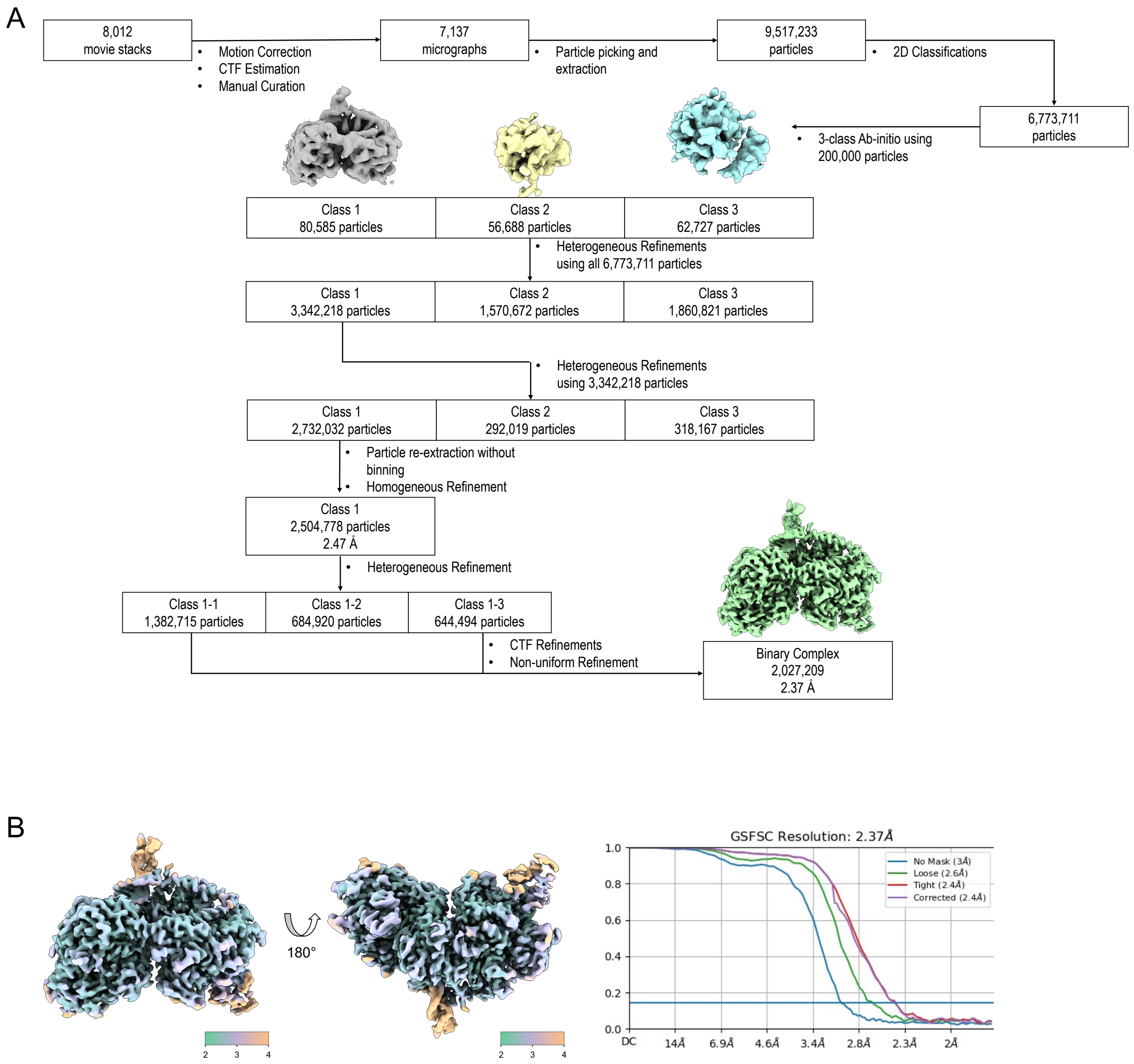

Figure S1 Cryo-EM workflow for wild-type Pol γ binary complex (A) CryoSPARC pipeline for structure determination. **(B)** Local resolution map of the wild-type Pol γ binary complex (left) and GSFSC plot (right).

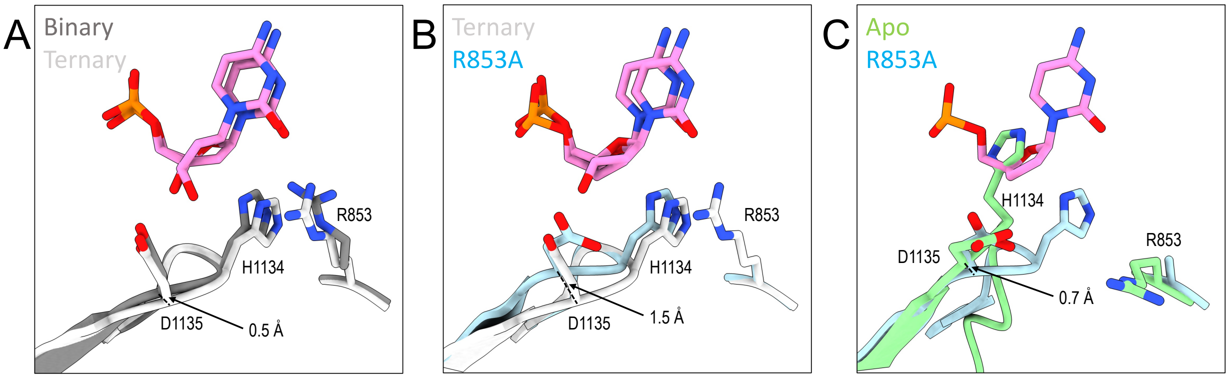

Figure S2 Comparison of catalytic loop in Pol γ Comparison of catalytic loop positioning relative to Arg^{853} and primer nucleotide (magenta) between **(A)** wild-type ternary (White) and binary (Gray) complexes, **(B)** wild-type ternary (White) and Pol γ R853A (Blue) ternary complexes, and **(C)** *apo* Pol γ (Green) and Pol γ R853A (Blue) ternary complex.

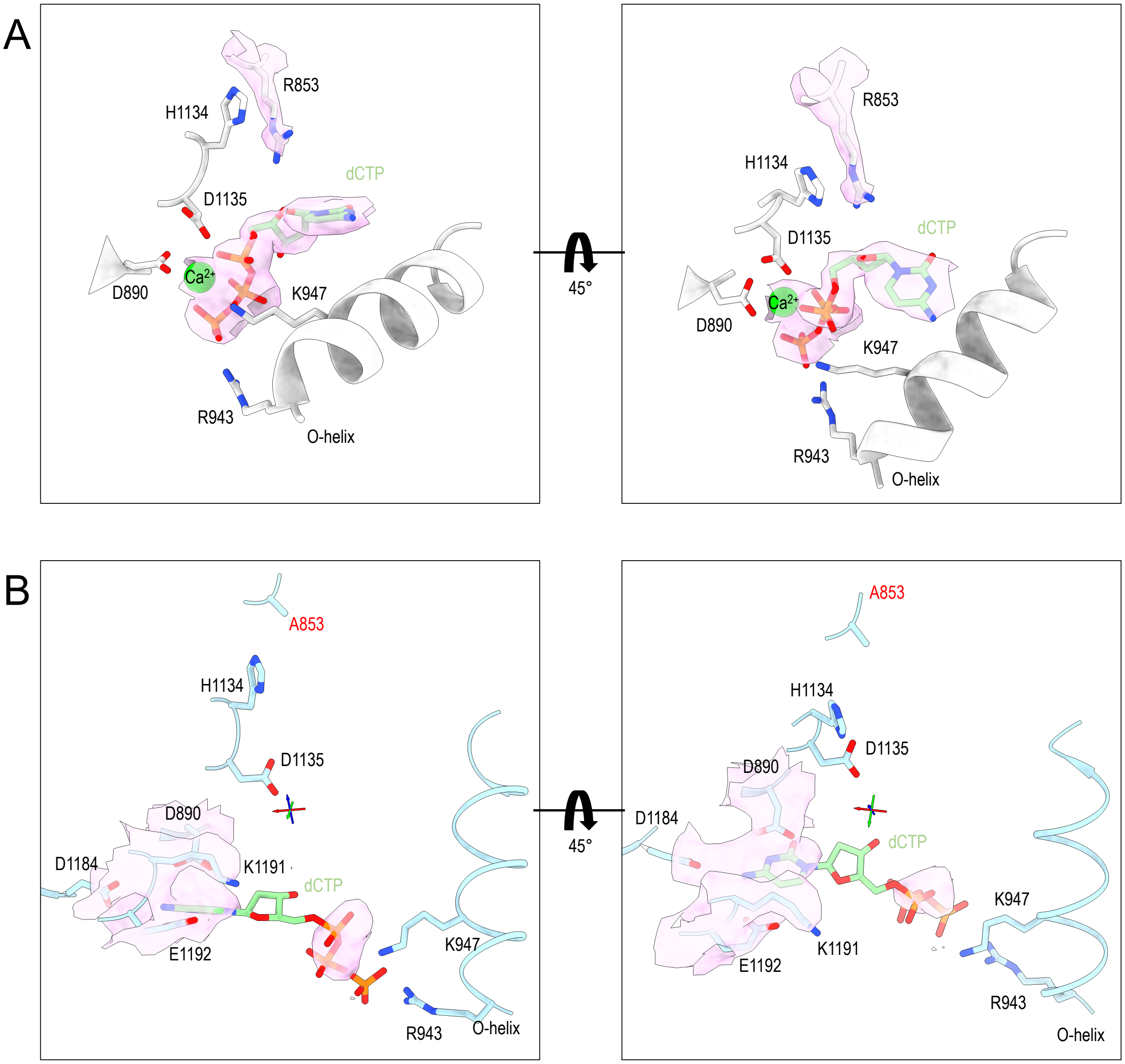

Figure S3 EM density in polymerase active site of wild-type and R853A Pol γ ternary complexes Polymerase active site of Pol γ with key interacting residues and dCTP is overlaid with isolated EM densities (Pink) in wild-type Pol γ **(A)** and Pol γ R853A **(B)** ternary complexes.

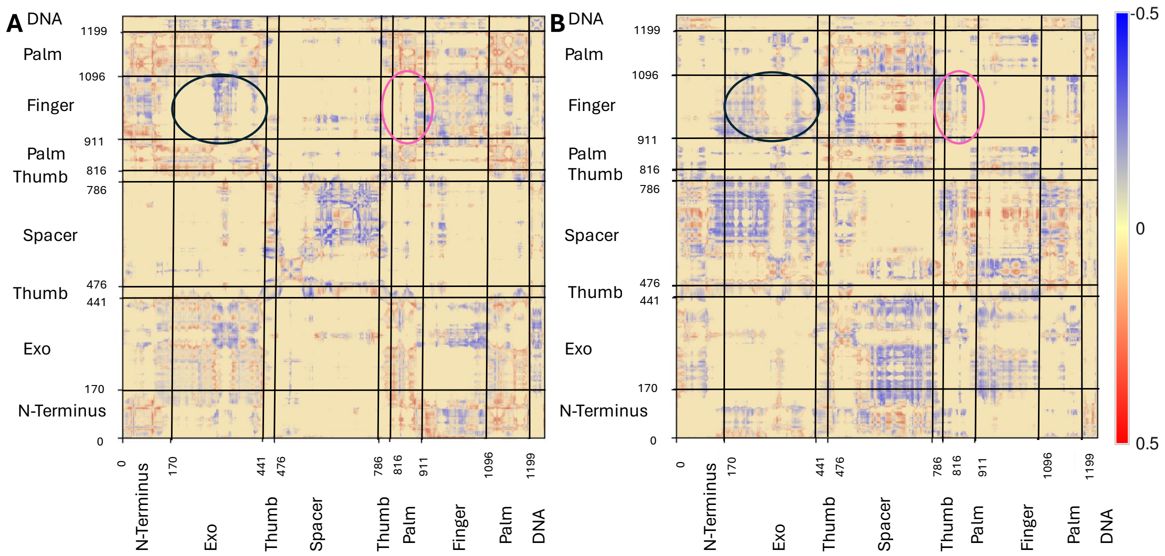

Figure S4 Dynamic cross correlation analysis of wild-type and R853A mutant

Dynamic cross correlation analysis of the mutant and the wild-type system showing the difference (MUT-WT) plots of the positive correlation values **(A)** and the difference (MUT-WT) of the negative correlation values **(B)**. It is seen that the interactions between the Palm and Fingers region (encircled in pink) shows anti-correlation motion in the wild-type, that is absent in the mutant (prevalent blue), which shows the mutation inhibits the open-close motion of the polymerase necessary for nucleotide incorporation, which is prevalent in the wild-type. It is also seen that the Fingers subdomain has decreased correlation with the Exo domain in the mutant as compared to the wild-type (encircled in black).

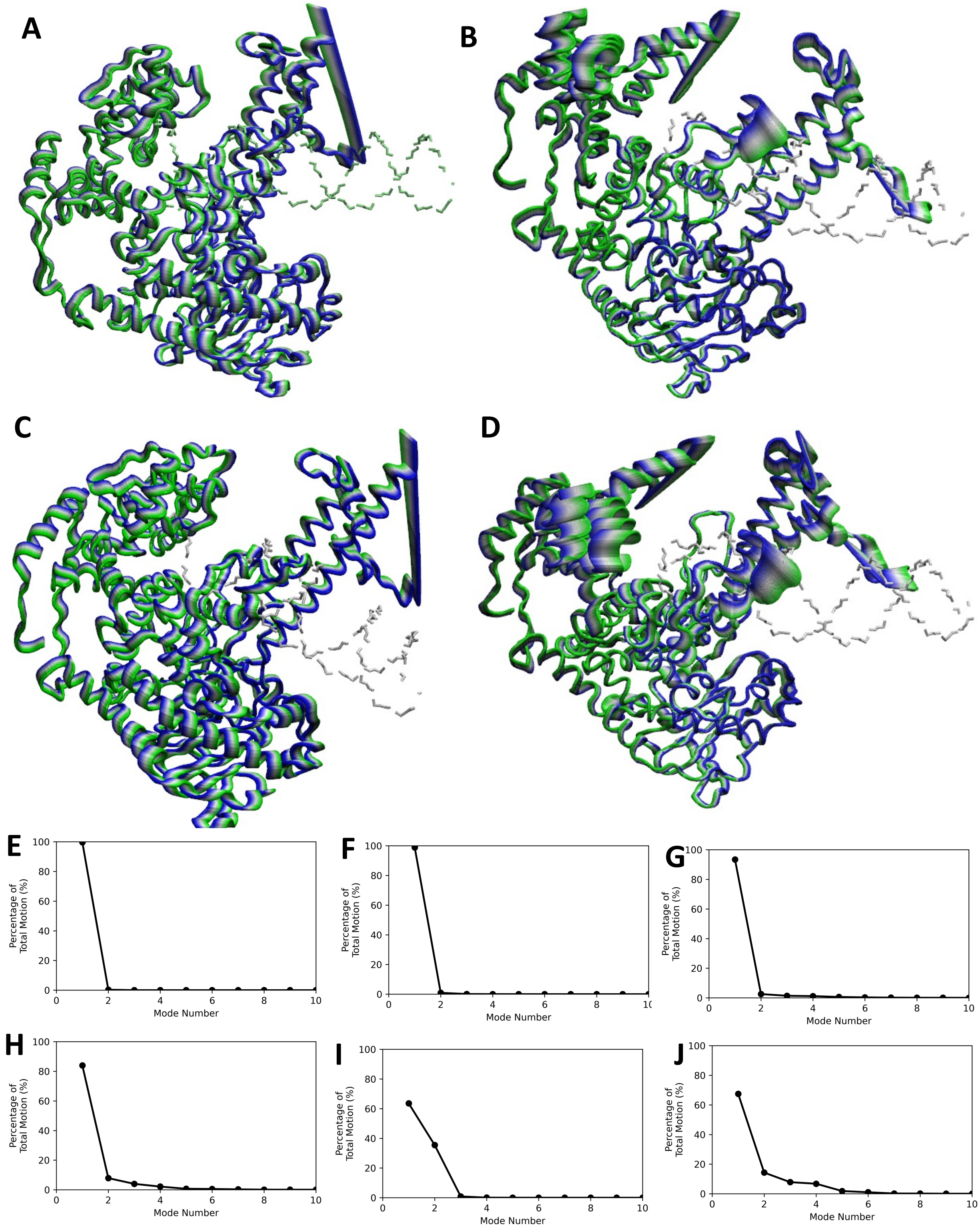

Figure S5 Normal Mode Analysis of wild-type and R853A mutant. Mode 1 of Normal Mode Analysis of wild-type **(A)** and mutant **(B)** and mode 2 of wild-type **(C)** and mutant **(D)** depicting breathing motion **(A and D)** and rocking motion **(B and C)**. Percentage

contribution of each mode to the dynamics of the system of each replicate of the wild-type **(E-G)** and R853A **(H-J)** systems.

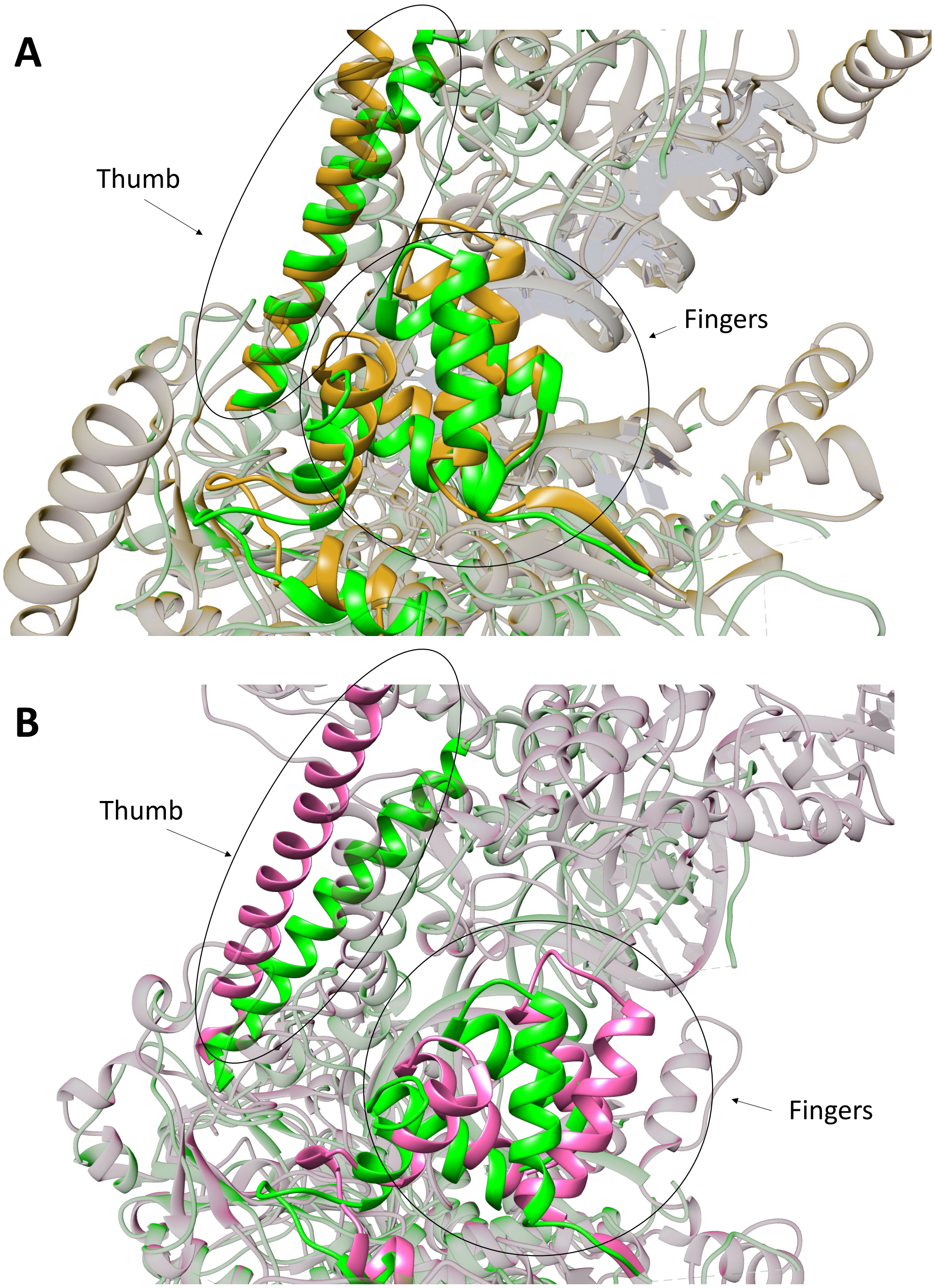

Figure S6 MD simulated structures of wild-type and mutant Pol γ The MD simulation resulted (A) wild-type Pol γ (gold) and (B) Pol γ R853A (pink) superimposed on the wild-type Pol γ ternary crystal structure (PDB: 4ZTZ, green). Thumb and Fingers subdomains are encircled for clarity.

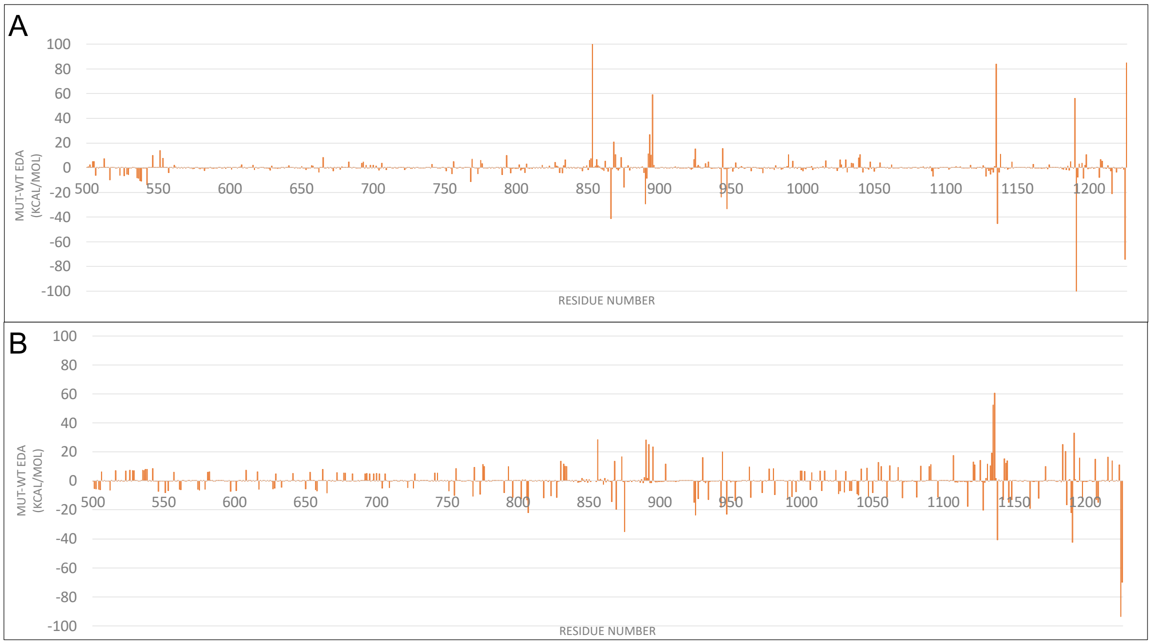

C

MUT-WT		MUT-WT	
W.R.T Incoming Nucleotide		W.R.T to the 853 residue site	
**Residues**	**EDA (kcal/mol)**	**Residues**	**EDA (kcal/mol)**
853R/A	123.86	875K	-35.02
866R	-41.23	890D	28.38
868D	20.68	892D	25.06
890D	-29.33	895E	23.54
893S	26.77	925K	-23.40
895E	59.18	947K	-23.02
943R	-23.78	1134H	19.21
947K	-33.17	1135D	52.37
1135D	83.93	1136E	60.53
1136E	-45.27	1138R	-40.56
1190R	56.19	1191K	-42.45
1191K	-125.07	1192E	33.14
1216E	-21.16	1225MeA	-93.70
1225 MeA	-74.22	1226 MeB	-69.88
1226 MeB	85.05	dCTP	123.86

Figure S7 Energy decomposition analysis Difference of energy decomposition analysis of MUT-WT with respect to dCTP **(A)** and with respect to mutation site, A.A. 853 **(B)**. **(C)** Table depicting the amino acids that show strong stabilizing/destabilizing interactions with respect to the incoming nucleotide and the mutation site.

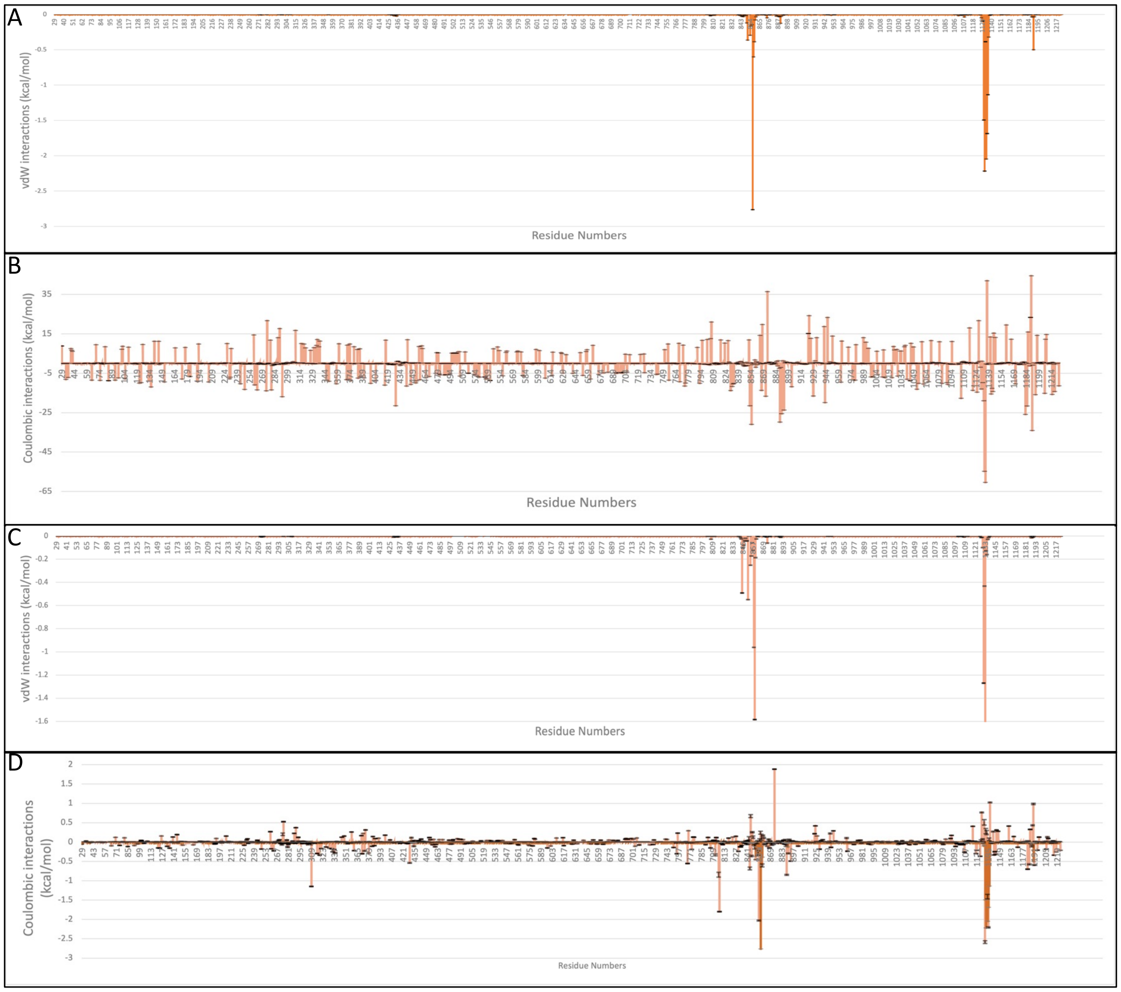

Figure S8 EDA separated into coulombic and van der Waals interactions for Wild-Type and Mutant representative replicates van der Waals and coulombic interactions for replicate 1 of Wild-Type **(A)** and **(B)** and of the R853A mutant **(C)** and **(D)** with respect to the residue site 853 showing error bars accounting for the 50000 sampled conformers for each system.

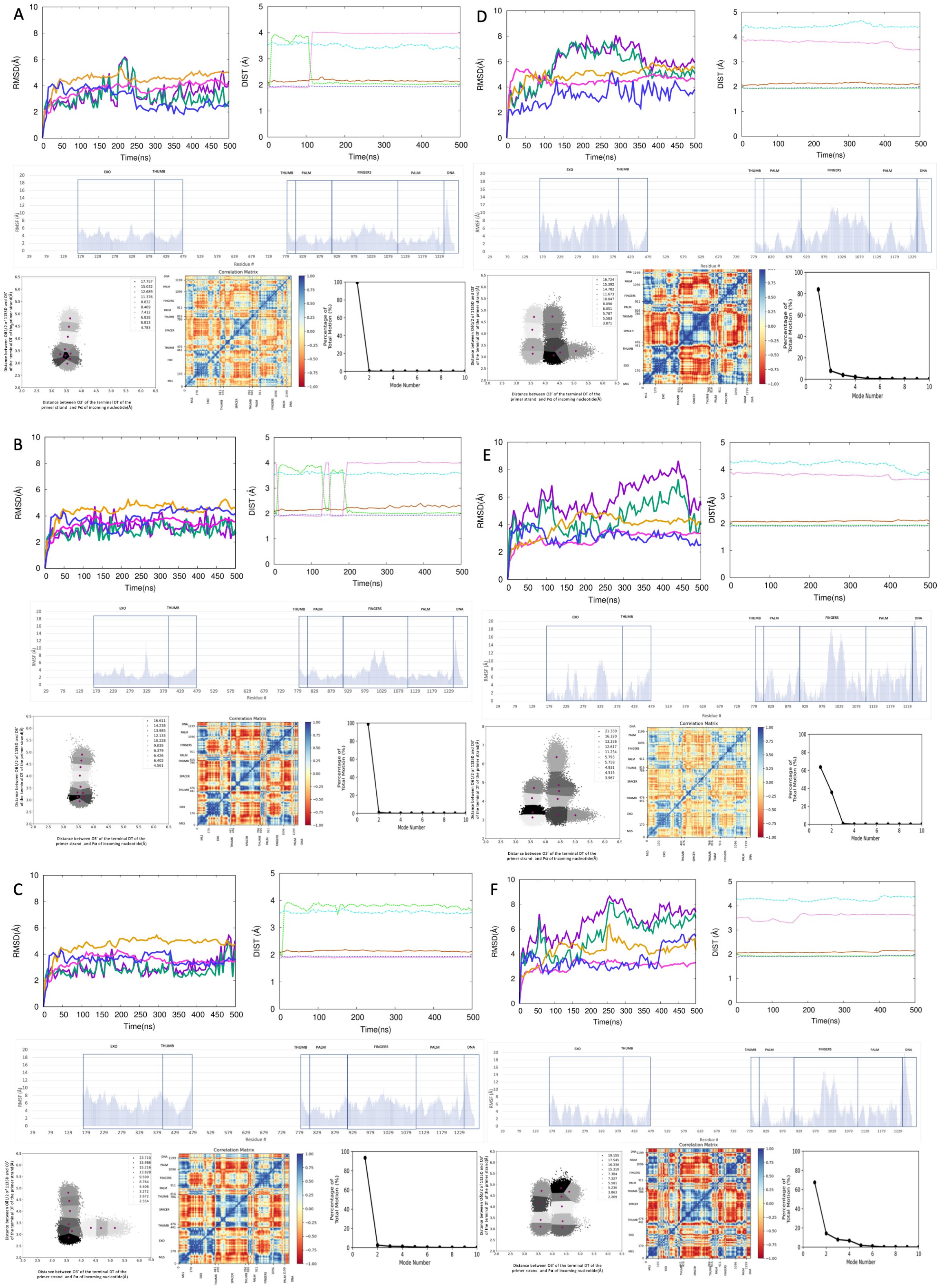

Figure S9 Triplicates of MD simulations of Pol γ wild-type and R853A Each panel for either wild-type (A-C) or Pol γ R853A (D-F) is arranged identically with RMSD analysis on top right, Distance analysis on the top left, K-means clustering, based on the distance (Å) between

the Oδ1/2 of 1135D and the O3' of the terminal primer DT, on the bottom left where less than 8% of the total sampled structures are located in the side tail in all repeats, and greater than 90% of the ensembles are clustered in the main body, with the percentage population of each cluster is given in top-right. \Correlation analysis on the bottom middle.

SUPPLEMENTARY TABLES

TABLE S1 MOLECULAR DYNAMICS SIMULATION STEPS AND RESTRAINTS

METHOD	NUMBER OF STEPS	RESTRAINT ON AS RESIDUES (kcal mol^{-1} $Å^{-2}$)	RESTRAINT ON REST OF PROTEIN (kcal mol^{-1} $Å^{-2}$)
Minimization	10000	100	100
Heating	500000	100	100
Equilibration	500000	100	100
Equilibration	500000	80	80
Equilibration	500000	60	60
Equilibration	500000	40	40
Equilibration	500000	20	20
Equilibration	500000	10	10
Equilibration	500000	5	5
Equilibration	500000	2	2
Equilibration	500000	1	1
Equilibration	500000	0.5	0.5
Equilibration	12500000	0.5	0
Production	250000000	0	0

Movies S1 WT and R853A normal mode analysis animation comparison
